# Evaluation of the efficacy and safety of first- and second-line immunotherapy in patients with metastatic colorectal cancer: a systematic review and network meta-analysis based on randomized controlled trials

**DOI:** 10.3389/fimmu.2024.1439624

**Published:** 2024-09-18

**Authors:** Kaiqi Chen, Wei Chen, Rui Yue, Danping Zhu, Shikui Cui, Xijian Zhang, Zhao Jin, Tong Xiao

**Affiliations:** ^1^ School of Basic Medical, Chengdu University of Traditional Chinese Medicine, Chengdu, China; ^2^ Department of Pharmacy, Emergency General Hospital, Beijing, China; ^3^ Department of Traditional Chinese Medicine, Chongqing Changhang Hospital, Chongqing, China; ^4^ Department of Endocrinology, Chongqing Hospital of Traditional Chinese Medicine, Chongqing, China; ^5^ School of Basic Medical Sciences, Capital Medical University, Beijing, China

**Keywords:** metastatic colorectal cancer, first- and second-line, immunotherapy, efficacy and safety, network meta-analysis, microsatellite status

## Abstract

**Background:**

A multitude of randomized controlled trials (RCTs) conducted in both the initial and subsequent treatment settings for patients diagnosed with metastatic colorectal cancer (mCRC) have provided clinical evidence supporting the efficacy of immunotherapy with the use of immune checkpoint inhibitors (ICIs). In light of these findings, the U.S. Food and Drug Administration (FDA) has authorized the use of several ICIs in specific subpopulations of mCRC patients. Nevertheless, there remains a dearth of direct comparative RCTs evaluating various treatment options. Consequently, the most effective ICI therapeutic strategy for microsatellite-stable (MSS) subgroup and microsatellite instability (MSI) subgroup in the first- and second-line therapies remains undefined. To address this gap, the present study employs a Bayesian network meta-analysis to ascertain the most effective first- and second-line ICI therapeutic strategies.

**Methods:**

A comprehensive literature search was conducted across multiple databases, including PubMed, EMBASE, Cochrane Library, and Web of Science, with the retrieval date ranging from the databases’ inception to August 20, 2024. A total of 875 studies were identified, and seven were ultimately included in the analysis after a screening process. A systematic review and network meta-analysis were conducted on the basis of the search results.

**Results:**

This comprehensive analysis, comprising seven RCTs, evaluated first-line and second-line immunotherapy regimens in 1,358 patients diagnosed with mCRC. The treatments under investigation consisted of five initial treatments, including three focusing on MSS patients and two on MSI patients, as well as two secondary immunotherapy regimens, both focusing on MSS patients. A total of 1051 individuals underwent first-line treatment, while 307 received second-line treatment. The application of ICIs proved to offer varying degrees clinical benefits when compared to standard-of-care therapy alone, both in two subgroups of the first and the second treatment phases. Of particular note is the performance of Nivolumab combination with ipilimumab, which demonstrated superior efficacy in improving progression-free survival (PFS) (HR=0.21; 95% CI, 0.13-0.34),. Moreover, the treatment demonstrated an optimal safety profile, with a relatively low risk of adverse events (OR = 0.33; 95% CI, 0.19–0.56), compared to other first-line treatment modalities for MSI subgroup. Regarding MSS subgroup, the improvement of PFS by Nivolumab plus standard-of-care (SOC) was relatively significant (HR = 0.74; 95% CI, 0.53-1.02). In the realm of second-line therapies for MSS subgroup, the administration of Atezolizumab plus SOC has proven to be an effective approach for prolonging PFS, exhibiting an HR of 0.66 (95% CI, 0.44–0.99). These findings underscore the clinical benefits and safety profiles of ICIs in the treatment of mCRC across various treatment lines.

**Conclusions:**

The clinical application of ICIs in both first- and second-line treatment strategies for patients with mCRC yields substantial therapeutic benefits. A detailed assessment in this study indicates that first-line treatment with Nivolumab combination with ipilimumab may represent an efficacious and well-tolerated therapeutic approach for MSI subgroup. In terms of MSS subgroup in first-line therapy, Nivolumab plus SOC may be a relative superior choice. In the context of second-line therapy for MSS subgroup, it is evident that a combination of Atezolizumab and SOC represents a preferable option for enhancing PFS. Furthermore, it is noteworthy that other ICIs treatment regimens also exhibit great value in various aspects, with the potential to inform the development of future clinical treatment guidelines and provide a stronger rationale for the selection of ICIs in both first- and second-line therapeutic strategies for mCRC.

**Systematic review registration:**

https://www.crd.york.ac.uk/prospero/#recordDetails, identifier CRD42024543400.

## 1 Introduction

Colorectal cancer represents the third most prevalent gastrointestinal malignancy globally and is the second leading cause of cancer-specific deaths (1). By 2035, the estimated number of new colorectal cancer cases worldwide is anticipated to reach approximately 2.5 million (2), posing a considerable threat to public health. Notably, above 20% of colorectal cancer patients already exhibit distant metastases upon initial diagnosis (3, 4), and the five-year survival rate being less than 20% (5). It is particularly disappointing that fewer than 20% of patients with mCRC are able to achieve a cure through surgical resection (6). In patients with unresectable tumors, systemic therapy rooted in chemo regimens such as CAPOX and FOLFOX prevails as the most efficacious treatment strategy (7). However, chemotherapy’s efficacy, while commendable in managing mCRC, fails to substantially elevate two-year survival rates, underscoring the generally bleak prognosis (8). Consequently, chemotherapy alone appears limited in its potential for mCRC. Recent advancements have introduced targeted therapies like cetuximab, panitumumab, aflibercept, ramucirumab and bevacizumab, when coupled with chemotherapy, demonstrating enhanced efficacy in mCRC clinical trials. Notably, these combinatorial approaches have protracted OS from 16-19 months to over 30 months (9), with bevacizumab specifically exhibiting improvement in ORR, PFS, and OS (10). Nonetheless, the five-year survival rate remains largely unaltered (11), and targeted therapies confront constraints attributed to target specificity limitations and drug resistance (12).

The advent of immunotherapy has ushered in a new era in cancer treatment (13), particularly in the management of mCRC, which has attracted considerable attention in this field. Nevertheless, recent studies have demonstrated that immune heterogeneity has a significant impact on treatment sensitivity (14), and the efficacy of ICIs is intricately intertwined with the patient’s microsatellite status, resulting in considerable variations in immunotherapy response among different CRC cases (3). MCRC can be divided into two categories: microsatellite-stable (MSS) and microsatellite instability (MSI). Due to the distinct biological characteristics of these two microsatellite statuses, they exhibit different responses in immunotherapy. MSI accounts for approximately 10% of mCRC patients. MSI-type colorectal cancer possesses a large number of tumor-specific neoantigens and a higher level of tumor mutation burden, making it easier to activate the immune system and generate an anti-tumor response (15). It is notable that PD-1/PD-L1 inhibitors have exhibited promising results in the management of the majority of cases of microsatellite instability-high (MSI-H) or mismatch repair-deficient (dMMR) mCRC. For instance, in the KEYNOTE-177 trial (16), pembrolizumab doubled the median PFS of patients compared to chemotherapy. In the CheckMate-142 trial (17), the combination of nivolumab and ipilimumab significantly improved the ORR and complete response (CR) of patients. The observed phenomenon can be attributed to the fact that mCRC patients with high MSI levels typically present a tumor microenvironment distinguished by T-cell infiltration (18), which is associated with reduced responsiveness to fluorouracil-based chemotherapy yet increased sensitivity to PD-1 monoclonal antibody therapy (19), so that immunotherapy is more effective in such patients. In contrast, MSS accounts for approximately 90% of MCRC cases. Due to its lower mutation burden, there are more immunosuppressive cells such as regulatory T cells and myeloid-derived suppressor cells in the tumor microenvironment, leading to poor response to immunotherapy (20). In the cases of MSS or pMMR mCRC patients, the majority of clinical trial outcomes over the past five years have been disappointing (21). For instance, in the Keynote-016 clinical trial (22), none of the 18 patients exhibited an ORR upon administration of Pembrolizumab. Likewise, in a separate study encompassing 73 mCRC patients (23), no ORR was observed following combination therapy with Regorafenib and Pembrolizumab. This suggests that ICIs fail to achieve satisfactory clinical efficacy for the majority of MSS and MSI-L mCRC patients (24). Accordingly, the prevailing theory is that a ‘cold’ microenvironment is responsible for this phenomenon. It is the ‘cold’ microenvironment that impedes the optimal effectiveness of immunotherapy with ICIs. Nevertheless, there is also evidence indicating that ICIs may be a potential therapeutic option for MSS/pMMR mCRC patients (25), and the American Association for Cancer Research (AACR) in 2024 announced the results of NCT03711058, demonstrating that the combination of copanlisib and nivolumab can elicit durable responses in MSS colorectal cancer patients. In conclusion, the therapeutic potential of ICIs in the context of mCRC with varying microsatellite statuses remains open to question and therefore worthy of further investigation.

Although a number of RCTs have been conducted to evaluate the efficacy of ICIs, there remains considerable debate regarding the optimal regimens for such treatments due to the scarcity of RCTs directly comparing different immunotherapies. This debate includes the question of which immunotherapy regimen is more effective for mCRC patients in the MSI and MSS subgroups, respectively. In order to address this issue, we have conducted systematic reviews and Bayesian network meta-analyses to evaluate and rank the effectiveness and safety of different immunotherapy strategies. Furthermore, we have performed separate analysis for first- and second-line immunotherapy, offering a nuanced discussion on subgroups with different microsatellite statuses. This study aims to offer valuable evidence-based medical insights to assist with clinical decision-making.

## 2 Materials and methods

### 2.1 Data sources and search strategy

A systematic search of the PubMed, EMBASE, Cochrane Library, and Web of Science databases was conducted up to August 20, 2024, using a combination of free-text and subject terms. The keywords included “Metastatic Colorectal Cancer,” “randomized clinical trial,” “immune checkpoint inhibitors,” “PD-L1 inhibitor,” “PD-1 inhibitor,” and “CTLA-4 Inhibitor.” To ensure transparency, reliability, and originality, the study protocol has been prospectively registered in the International Prospective Register of Systematic Reviews (PROSPERO). This registration number is CRD42024543400.

### 2.2 Selection criteria

Inclusion criteria:

1. RCTs involving histologically or cytologically confirmed patients with mCRC are included.
2. RCTs utilizing ICIs as first-line or second-line therapeutic regimens for mCRC patients are considered.
3. RCTs that compare the efficacy of ICIs with standard treatment protocols as first-line or second-line therapies for mCRC are included.
4. RCTs must report at least one of the following outcome measures: OS, PFS, ORR, and the incidence of grade 3 or higher adverse events.

Exclusion criteria:

1. RCTs based on the same cohort of patients but conducted at different stages are excluded to avoid duplication.
2. RCTs with unclear or ambiguous outcome measures are not included.
3. Reviews or case reports are excluded from this analysis.

### 2.3 Data extraction and quality assessment

Two researchers independently extracted data from the RCTs in accordance with the PROSMA statement. Any discrepancies were resolved through discussion with a third author. From each article, the following information was extracted: trial name, trial design, publication source, publication year, tumor stage, national clinical trial number, sample size, and dosing regimens for both the experimental and control groups. The outcome measures extracted from each article included Hazard Ratio (HR) for PFS, along with their corresponding 95% confidence intervals (95% CI), Odds Ratio (OR) for adverse events (AEs) of grade 3 or higher. The quality of the included RCTs was assessed using the Cochrane Risk of Bias Tool (2.0). This evaluation tool is based on the following five domains: risk of bias arising from the randomization process, risk of bias due to deviations from the intended interventions, risk of bias from missing outcome data, risk of bias in the measurement of the outcome, and risk of bias in the selection of the reported result. The risk assessment ratings for the included RCTs were categorized into three levels: low risk, high risk, and having “some concerns”.

### 2.4 Statistical analysis

The primary endpoints were PFS, while the secondary endpoints included grade 3 or higher AEs. HR and 95% CI were used as effect sizes for PFS, whereas OR and 95% CI served as effect sizes for grade 3 or higher AEs.

A network meta-analysis was conducted within a Bayesian framework utilizing the “rjags” and “gemtc” packages in R software (26, 27). A fixed-effects model was employed, and three independent Markov chains were established, each running 10,000 burn-ins and 30,000 sample iterations. The iteration results of the Markov chains, using HR and OR as effect sizes, were used to rank the efficacy and safety of different treatment regimens, which were then visually presented.

This study also utilized Revman 5.4 software to conduct a pairwise meta-analysis based on the frequency method, providing an overall assessment of the efficacy and safety of first-line and second-line immunotherapy compared to standard treatments. Heterogeneity was assessed using the *Q* test and *I²* statistic, with *I²* ≤ 50% or *P* ≥ 0.1 indicating low heterogeneity, and *I²* > 50% or *P* < 0.1 indicating high heterogeneity. For studies with high heterogeneity, a random-effects model was applied, while a fixed-effects model was used for studies with low heterogeneity. For studies with high heterogeneity, sensitivity analysis was performed, and studies with significant impacts on heterogeneity were sequentially excluded from the model.

### 2.5 Sensitivity analysis

Using the Deviance Information Criterion (DIC) for model comparison, we evaluate the relative goodness-of-fit between the fixed-effects model and the random-effects model. A smaller DIC value indicates a better model fit. If the DIC difference between the fixed-effects model and the random-effects model is less than 5, the models are considered to be consistent.

## 3 Results

### 3.1 Study selection

A total of 875 studies were screened, and after excluding duplicates and irrelevant studies based on titles and abstracts, we conducted a detailed review of the remaining 78 studies eligible for full-text examination. Among them, 71 were further excluded for the following reasons that were not in line with the inclusion criteria, including that the experimental group and the control group did not use different drugs to evaluate the safety and efficacy of ICIs compared with the standard treatment regimen, and the patient population belonged to the subgroup of other classification criteria (such as RAS mutation).Ultimately, we selected 7 studies (16, 28–33), all of which were randomized controlled trials involving 1358 eligible patients. Among them, 1051 patients received the following five first-line treatments: standard-of-care therapy (SOC), Pembrolizumab (Pem), Atezolizumab plus standard-of-care therapy (Ate-SOC), Nivolumab plus ipilimumab (Niv-ipi) and Nivolumab plus standard-of-care therapy (Niv-SOC). Additionally, 307 patients underwent the following three second-line treatment regimens: standard-of-care therapy (SOC), Tremelimumab plus Durvalumab (Tre-Dur), and Atezolizumab plus standard-of-care therapy (Ate-SOC).

**Figure 1:**
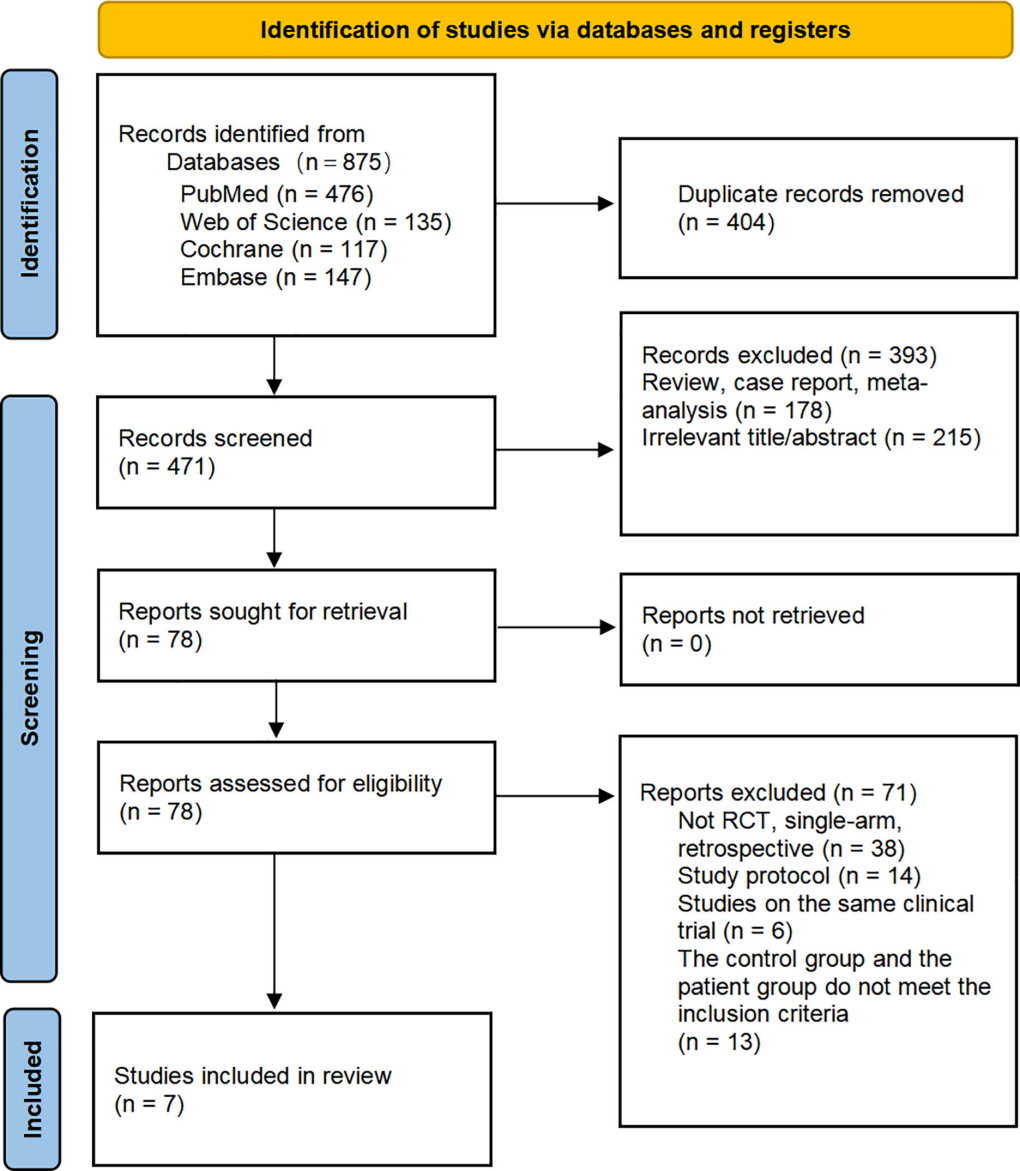
Flow chart of included and excluded studies.

**Figure 2:**
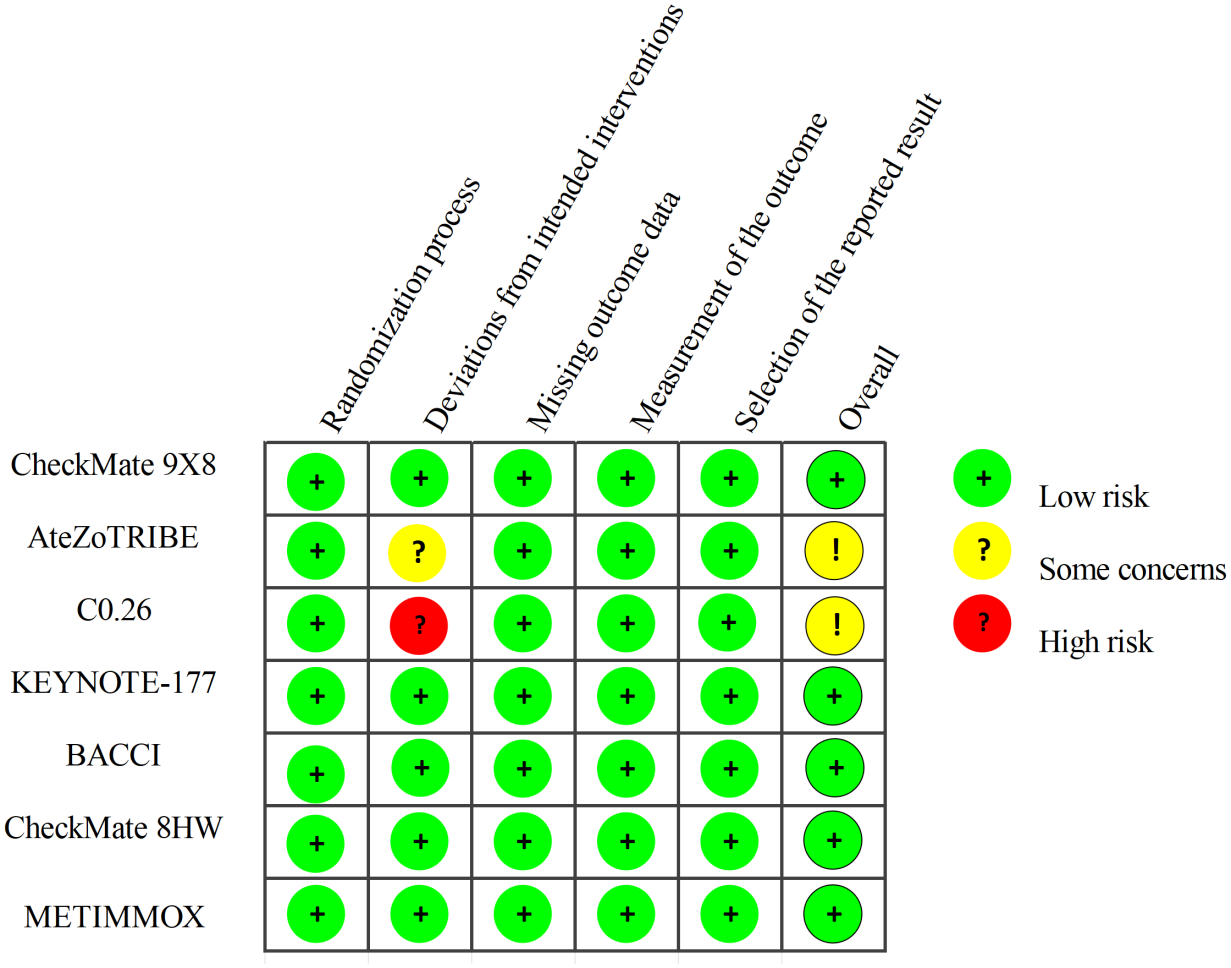
Risk of bias summary.

**Figure 1** provides detailed information on the literature search conducted in this study. **Table 1** outlines the basic characteristics of the included studies. **Figure 2** presents the quality assessment of the included studies, which are all moderately or highly credible. However, one study exhibits high risk and another study with potential problems deserves attention.

**Table 1:** Basic characteristics of included studies.

Author (Year)	Study	Registered ID	Sample size	Included sample	stage	Microsatellite state(MSS/MSI)	Gender(M/F)	Intervention arms	Control arms	Primary end points
**Lenz (** 28) (2022)	**CheckMate9×8** **(phase2)**	NCT03414983	310	195(127/68)	first-line	(121/6)/(61/7)	119/76	Nivolumabplusstandard-of-caretherapy 240mg(Q2W)	standard-of-caretherapy (mFOLFOX6plusBEV)	PFS
**Antoniotti (** 29) (2022)	**AtezoTRIBE** **(phase2)**	NCT03721653	218	218(145/73)	first-line	(132/8)/(67/5)	125/93	Atezolizumabplusstandard-of-caretherapy 840mg(Q2W)	standard-of-caretherapy (FOLFOXIRIplusBEV)	PFS
**Diaz (** 16) (2022)	**KEYNOTE-177** **(phase3)**	NCT02563002	307	307(153/154)	first-line	(0/153)/(0/154)	153/154	Pembrolizumab 200mg(Q3W)	standard-of-caretherapy (mFOLFOX6plusBEV/FOLFIRIplusBEV/CET)	PFS
**Ree (** 30) (2024)	**METIMMOX** **(phase2)**	NCT03388190	80	76(38/38)	first-line	(38/0)/(38/0)	41/35	Nivolumabplusstandard-of-caretherapy 240mg(Q2W)	standard-of-caretherapy FLOX	PFS
**Lenz (** 31) (2024)	**CheckMate8HW** **(phase3)**	NCT04008030	303	255(171/84)	first-line	(0/171)/(0/84)	–	Nivolumabplusipilimumab 240mg(Q2W) 1mg/kg(Q3W)	standard-of-caretherapy	PFS
**Chen (** 32) (2020)	**CO.26** **(phase2)**	NCT02870920	180	180(118/61)	second-line	(117/1)/(49/1)	121/59	TremelimumabplusDurvalumab 75mg(Q4W) 1500mg(Q4W)	bestsupportivecare	PFS
**Mettu (** 33) (2022)	**BACCI** **(phase2)**	NCT02873195	133	128(82/46)	second-line	(69/6)/(41/3)	77/51	Atezolizumabplusstandard-of-caretherapy 1200mg(Q3W)	placeboplusstandard-of-caretherapy BEVplusCAP	PFS

PFS, progression-free survival; OS, overall survival; BEV, Bevacizumab; CET, Cetuximab; CAP, Capecitabine.

### 3.2 Pairwise meta-analysis

#### 3.2.1 Comparisons of PFS

A total of seven studies were included (16, 28–33), all of which reported PFS. In first-line treatment, these studies comprised four MSI subgroup treatments (Ate-Soc, Niv-Ipi, Pem, SOC). And three MSS subgroup treatments were included: Ate-Soc, Niv-Soc, SOC. A high degree of statistical heterogeneity was observed across the studies (P>0.1, I²=81%) and therefore a random-effect model was applied in order to conduct the meta-analysis. The findings indicated that the use of ICIs in first-line therapy was associated with a significant improvement in PFS (HR=0.51, 95% CI: 0.33-0.95).

In the MSI subgroup, ICIs demonstrated a significant advantage in prolonging PFS compared with SOC (HR=0.28, 95% CI: 0.11-0.72), but there was high heterogeneity among studies (P<0.1, I²=87%). Therefore, the source of heterogeneity was investigated in this subgroup, and it was found that in the AtezoTRIBE study, only 13 MSI patients were included (8 in the experimental group and 5 in the control group), with an HR value significantly lower than that in other studies, which was likely to be the major cause of heterogeneity. In the MSS subgroup, the application of ICIs also showed a certain degree of PFS benefit compared with chemotherapy (HR=0.75, 95% CI: 0.60-0.95), and there was no heterogeneity among studies (P>0.1, I²=0%).

**Figure 3:**
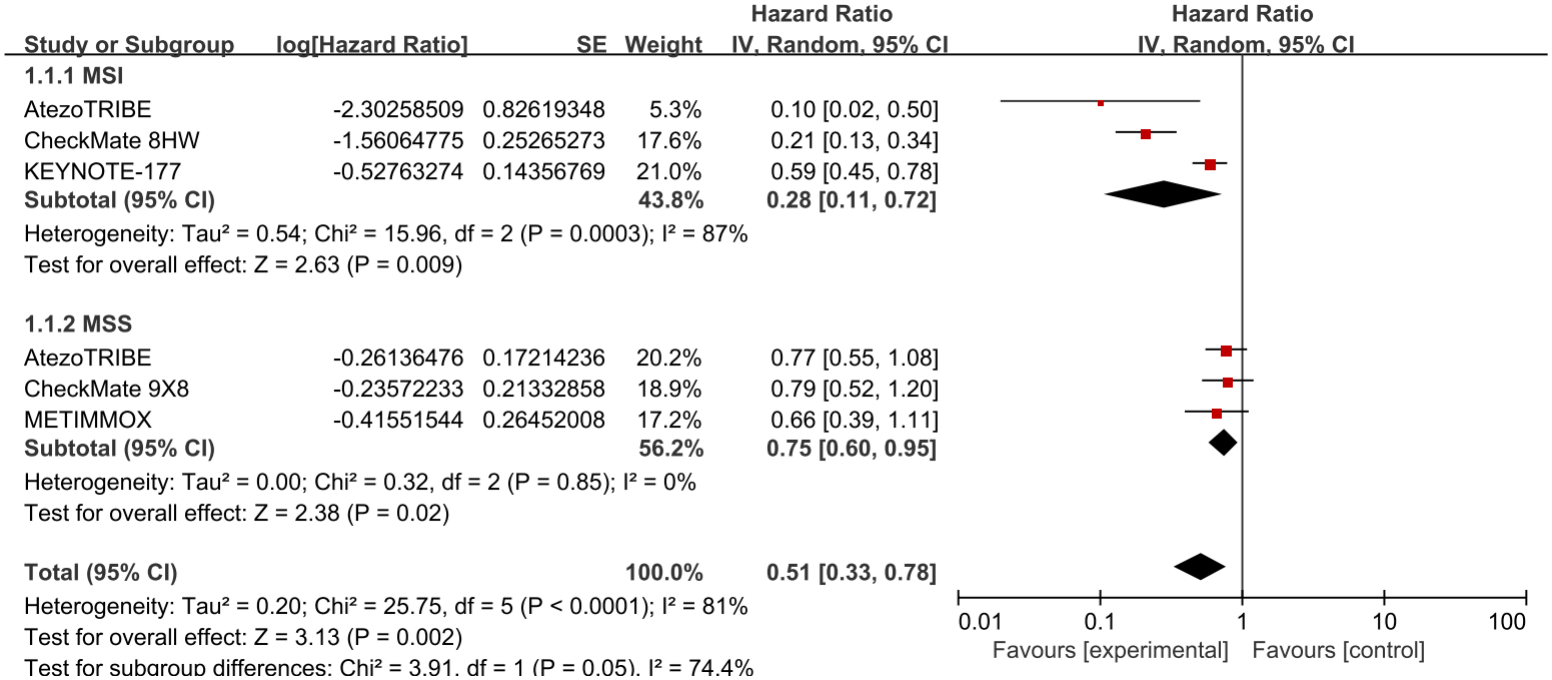
Comparison of PFS (first-line) between ICIs and SOC alone.

**Figure 4:**
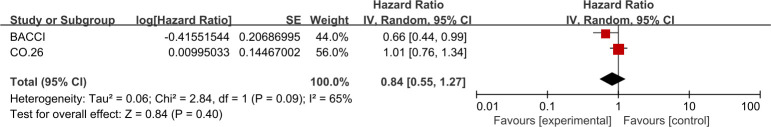
Comparison of PFS (second-line) between ICIs and SOC alone.

In second-line treatment, three MSS subgroup treatment regimens (Ate-SOC, Ter-Dur, SOC) were included. The heterogeneity of the studies was slightly high (P < 0.1, I² = 65), justifying the use of a random effects model for the meta-analysis. The findings revealed that ICIs in second-line treatment yielded marginally higher PFS benefits compared to SOC (HR=0.84, 95% CI: 0.55-1.27). A certain degree of heterogeneity was observed in these two studies, so the sources of heterogeneity were investigated. It was found that in the CO.26 study, due to the limitation of sample size, the PFS of the treatment group was 1.8 months and that of the control group was 1.9 months, which largely contributed to the generation of heterogeneity. See **Figures 3**, **4** for details.

#### 3.2.2 Comparisons of AEs≥3

**Figure 5:**
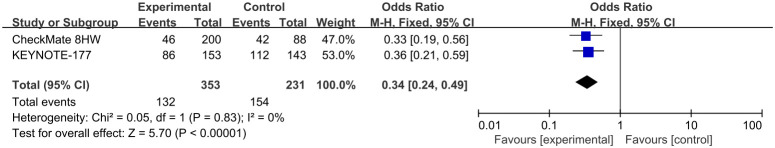
Comparison of safety between ICIs and SOC alone.

Two studies reported AE≥3 (13, 14, 32, 34–36), involving three first-line treatments for MSI subgroup (Pem, Niv-Ipi, SOC). Due to no statistical heterogeneity among studies (P>0.1, I²=0%), a fixed effects model was employed for meta-analysis. The results indicated that the risk of adverse events in patients receiving SOC alone in first-line treatment was marginally higher than that of ICIs(OR=0.34, 95% CI: 0.24-0.49). Details of this observation are presented in **Figure 5**.

### 3.3 Network meta-analyses

#### 3.3.1 Comparisons of PFS

**Figure 6:**
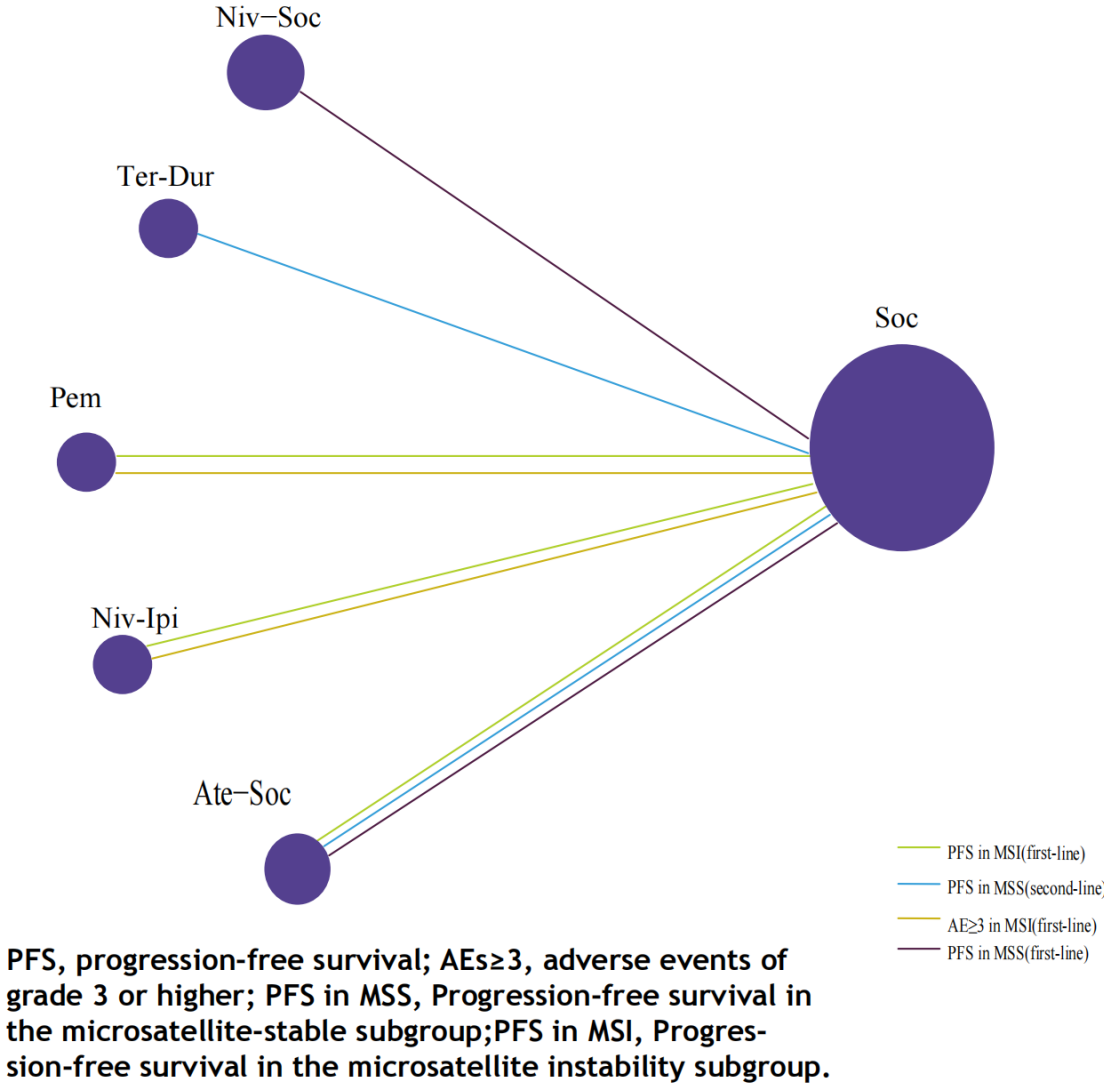
Network plot for endpoints of multiple ICIs regimens of metastatic colorectal cancer.

**Figure 7:**
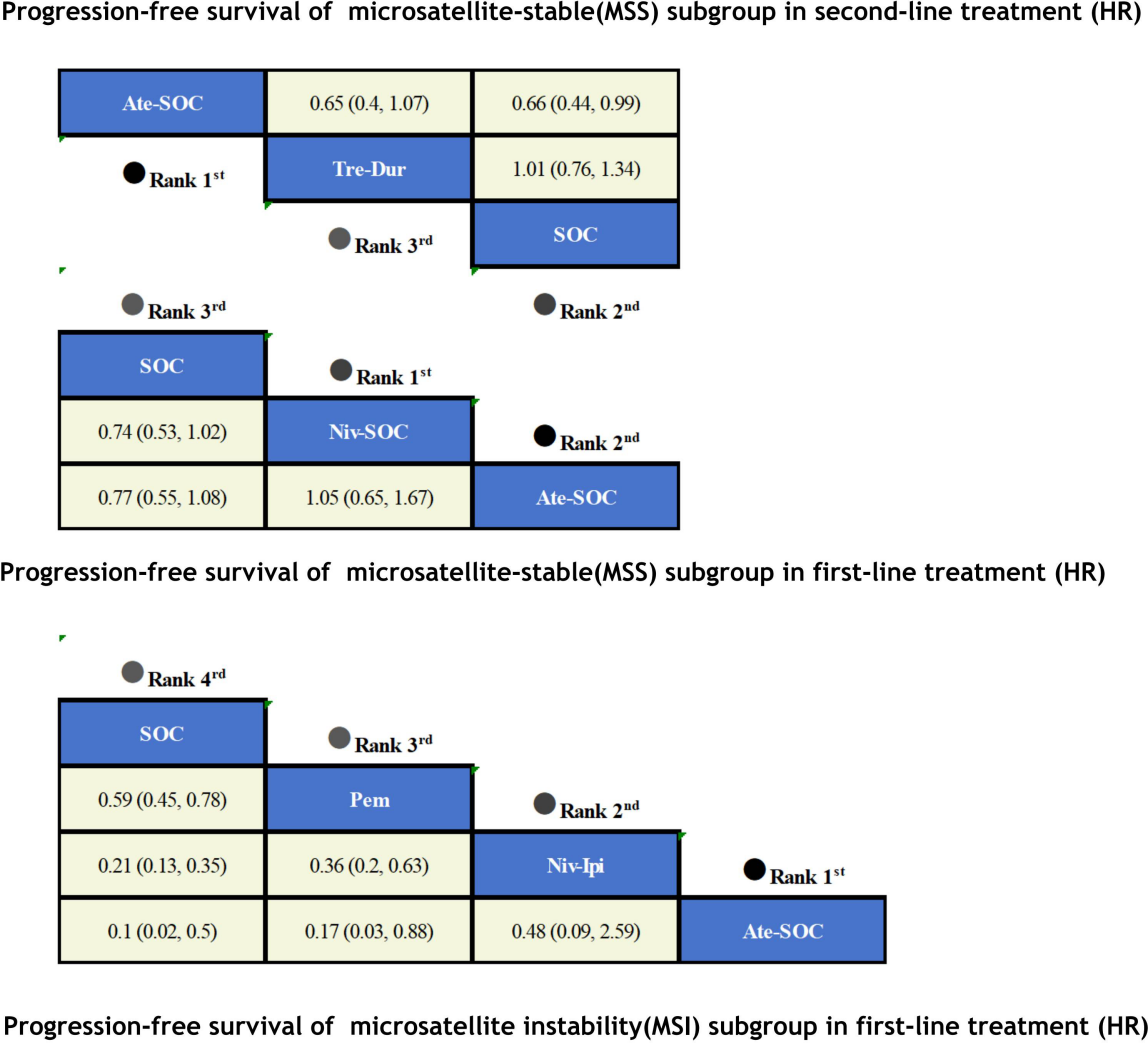
Summary treatment effects from the network meta-analysis for PFS.

The immunotherapy regimens included in the NMA reported PFS and AEs (**Figure 6**). Patients who received ICIs therapy exhibited longer PFS (**Figure 7**) compared to those who only received SOC. In the first-line setting, in the MSI subgroup, Ate-SOC was found to significantly enhance PFS (HR=0.10; 95% CI, 0.02-0.5). Subsequently, both Niv-Ipi (HR=0.21; 95% CI, 0.13-0.35) and Pem monotherapy (HR=0.59; 95% CI, 0.45-0.78) demonstrated advantages in improving PFS. In terms of PFS in the MSS subgroup, Niv-SOC demonstrated the most significant advantage in PFS benefit (HR=0.74; 95% CI, 0.53-1.02), followed by Ate-SOC (HR=0.77; 95% CI, 0.55-1.08). Compared to SOC alone, both combinations showed some improvement in PFS. In the MSI subgroup of second-line therapy, Ate-SOC exhibited the greatest clinical benefit in terms of PFS (HR=0.66; 95% CI, 0.44-0.99), with an estimated 12-month PFS rate of 15.2%, which was more than double that of the control group (6.9%). The least beneficial intervention with regard to improvement in PFS was Tre-Dur (HR = 1.01; 95% CI, 0.76–1.34), which was observed to be slightly less efficacious than the control group (SOC alone), with a median PFS of 1.8 months in the experimental group and 1.9 months in the control group.

#### 3.3.2 Comparisons of AEs≥3

**Figure 8:**
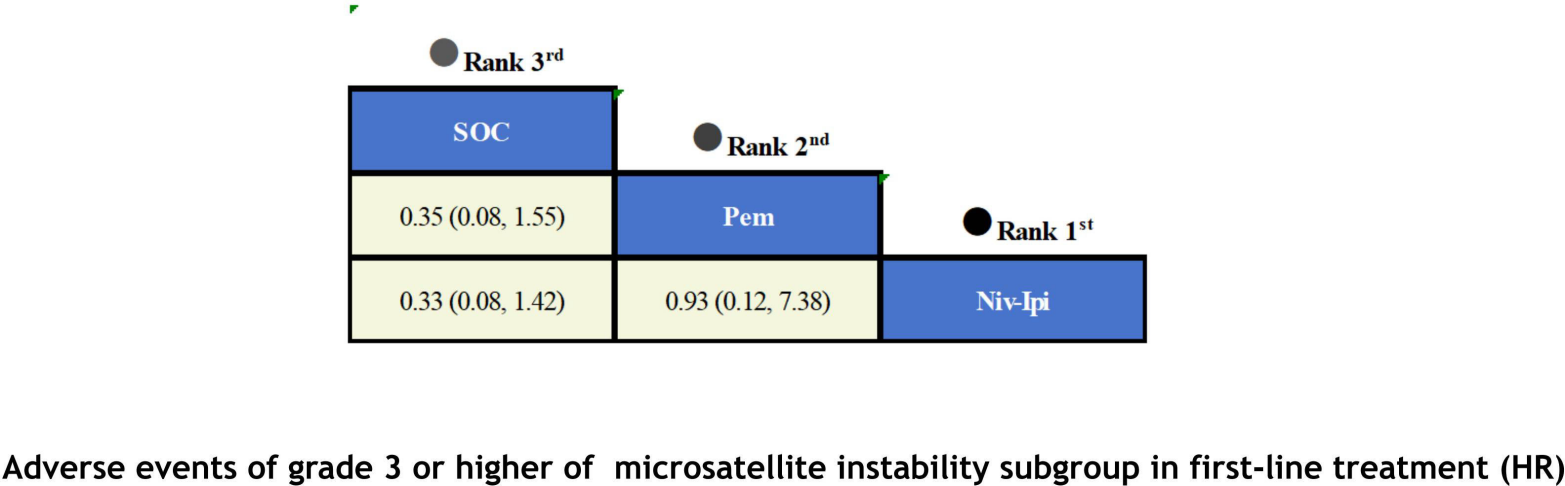
Summary treatment safety from the network meta-analysis for AEs≥3.

With regard to the matter of safety (**Figure 8**), due to data limitations in the original studies, we only included safety data from first-line treatment in the MSI subgroup for meta-analysis. Based on the available results, it has been observed that ICIs have not led to an increase in adverse events. Among patients receiving first-line therapy, the combination of Niv-Ipi demonstrated the highest safety (OR=0.33; 95% CI, 0.08-1.42), with only 23% of the experimental group reporting grade 3 or higher adverse events, compared to 48% in the control group. Following closely behind was Pem monotherapy (OR = 0.35; 95% CI, 0.08-1.55), which also exhibited a high level of safety, with 56% of the experimental group reporting grade 3 or higher adverse events and 78% in the control group.

### 3.4 Rankings

**Figure 9:**
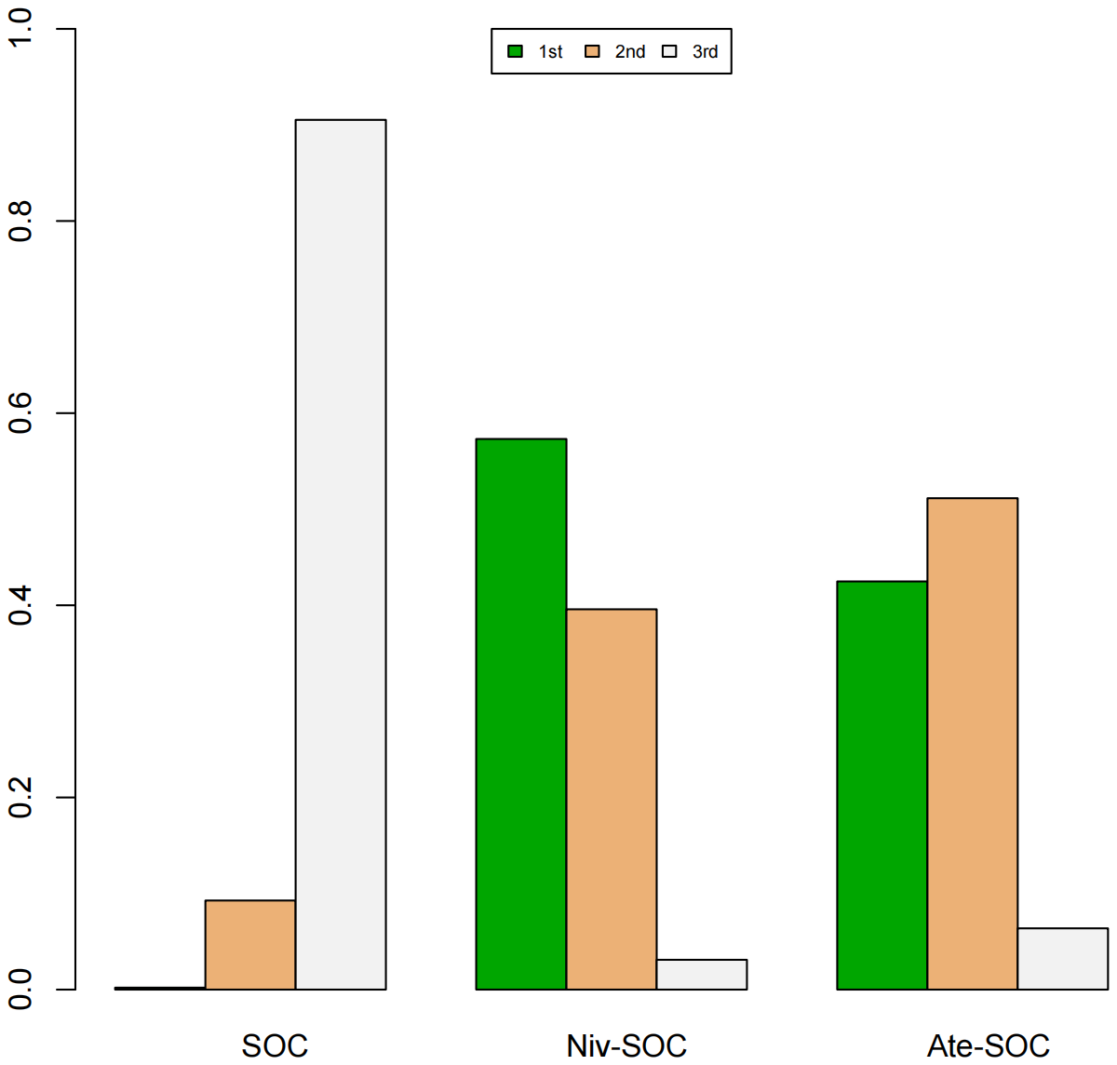
Ranking plot of PFS(MSS) in first-line treatment effects.

**Figure 10:**
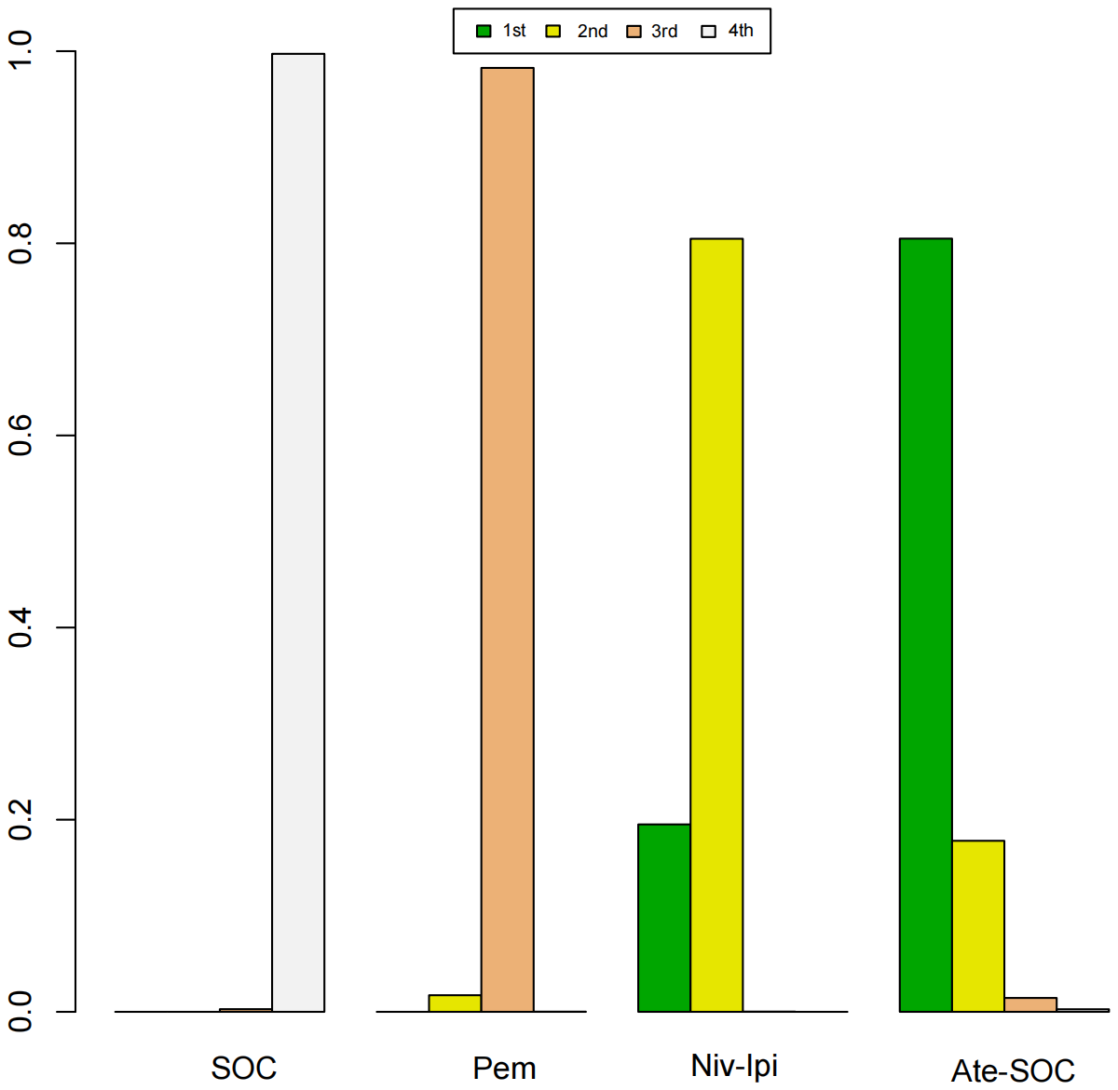
Ranking plot of PFS(MSI) in first-line treatment effects. Summary treatment effects from the network meta-analysis for ORR.

**Figure 11:**
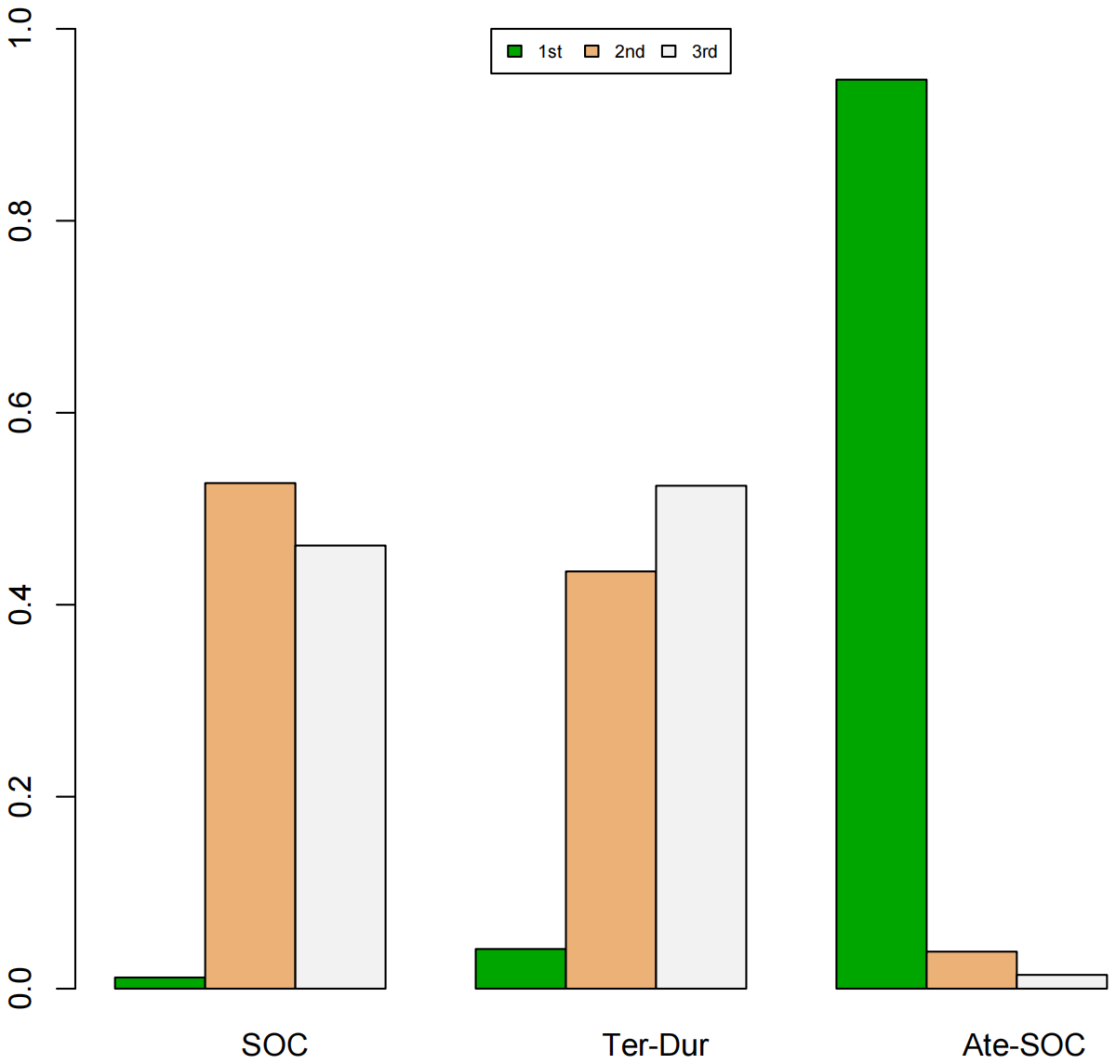
Ranking plot of PFS (MSS) in second-line treatment effects.

A Bayesian ranking spectrum analysis was conducted to evaluate the relative efficacy of various treatment options in mCRC patients. Regarding PFS, in the context of MSS subgroup in the first-line therapies, Niv-SOC emerged as the most probable candidate to rank first in improving PFS, with a 57% probability. This was followed by Ate-SOC, which had a 51% probability of ranking second. The probability of the SOC monotherapy ranking third was 90%. In MSI subgroup of the first-line treatments, Ate-SOC exhibited a 80% probability of being ranked first. The next treatment option was Niv-Ipi or Pem, with a 80% and 98% probability of being ranked second and third, respectively. The probability of SOC ranking fourth was 99%. In the MSS subgroup of second-line treatment, Ate-SOC demonstrated the highest probability of being ranked first, with an 94% likelihood. SOC and Ter-Dur were the next most probable, with 52% and 52% probabilities of ranking second and third, respectively. See **Figures 9**–**11** for details.

**Figure 12:**
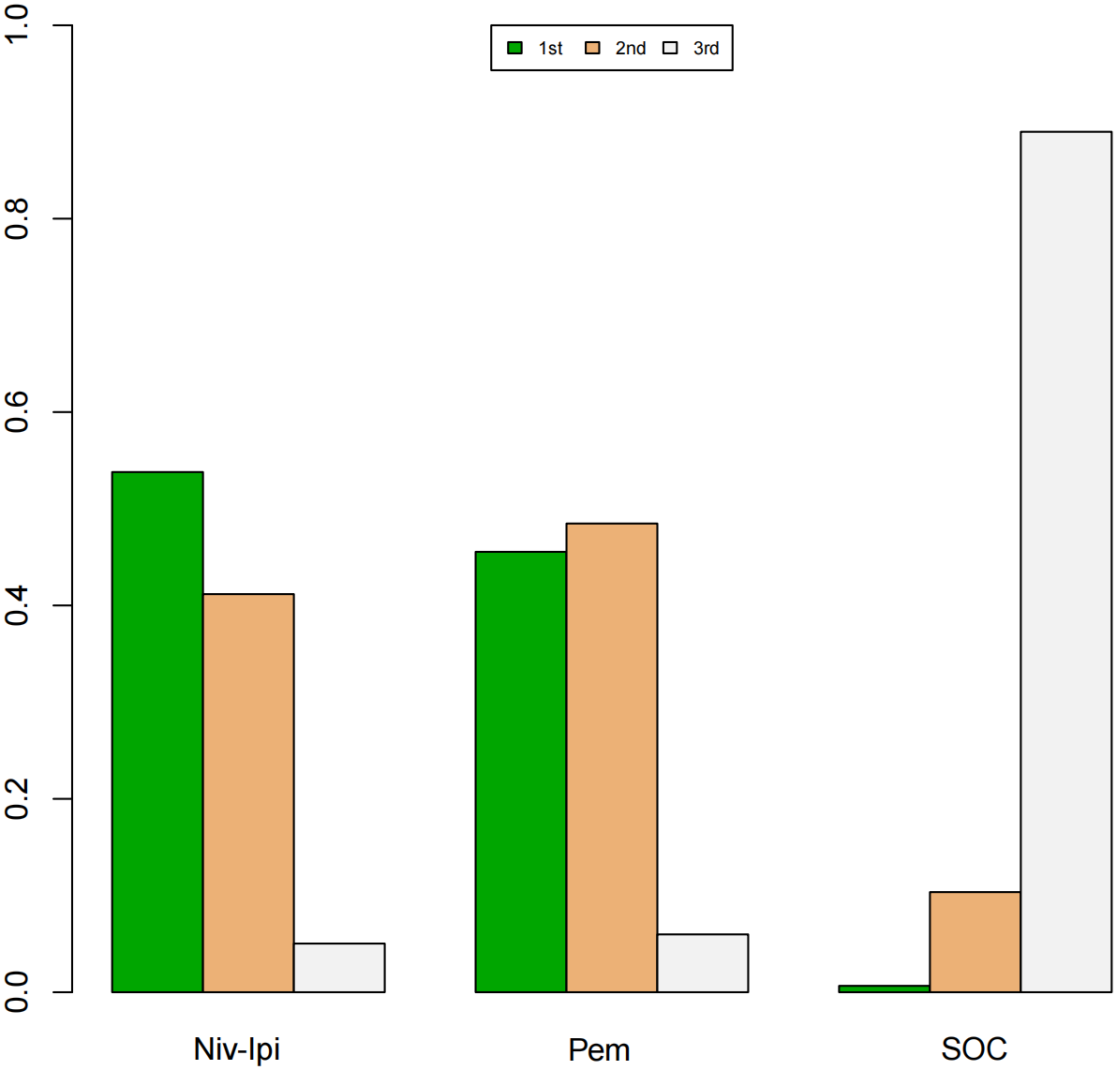
Ranking plot of AEs≥3(MSI) in first-line treatment safety.

Finally, in terms of safety, Niv-Ipi demonstrated the highest probability of being ranked first among first-line treatments, with an 53% likelihood. Pem and SOC were the next most probable, with 48% and 88% probabilities of ranking second and third, respectively. For further details, please refer to **Figure 12**.

## 4 Discussion

It is to our best knowledge that this study represents the first comprehensive evaluation of optimal immunotherapy strategies in first- and second-line treatments for mCRC. This study evaluated the efficacy and safety of ICIs, administered as monotherapy or in combination with SOC, across two dimensions. We employed an indirect rank order of all regimens in order to provide more reliable data for clinical applications. The analysis of seven studies, incorporating five different immunotherapy treatment protocols, indicated that both monotherapy with ICIs and combination therapy with SOC improved the therapeutic outcomes in mCRC patients as frontline treatment, regardless of whether they belong to the MSS or MSI subgroup. In terms of refractory populations, the application of ICIs in the MSS subgroup still presents some clinical benefits. Although our safety assessment of ICIs in frontline treatment is limited to the MSI subgroup due to the constraints of the original data, the results indicate that ICIs have reliable safety profiles. Specifically, the data demonstrated that ICIs treatment exhibited superior anti-tumor benefits. In terms of survival, Atezolizumab plus SOC demonstrated the most significant improvement in PFS in MSI subgroup of first-line therapy and MSS subgroup of second-line therapy. In MSS subgroup of first-line treatment, Nivolumab plus SOC achieved the best PFS benefit. Analyses of safety revealed that in first-line therapy Nivolumab combination with ipilimumab exhibited the most controllable safety and pembrolizumab monotherapy was also safer than SOC, demonstrating more reliable safety advantages over SOC alone.

Our study indicates that, in the initial treatment phase of MSI subgroup, the most optimal therapeutic strategy is the use of Nivolumab combination with ipilimumab, according to the current research data. This regimen achieved clinical benefits in PFS that were only slightly lower than those of Atezolizumab plus SOC, but with a more reliable sample size. Additionally, in terms of safety, Atezolizumab plus SOC lacks relevant subgroup data, whereas the combination of Nivolumab and ipilimumab exhibited the highest safety profile among the three regimens included in the meta-analysis. After comprehensive consideration, our study concludes that the combination of Nivolumab and ipilimumab may be the optimal current regimen for first-line treatment in the MSI subgroup. This conclusion diverges from previous research findings (34), which suggested that Pembrolizumab demonstrates notable clinical advantages and ought to be considered the foremost choice for initial treatment. However, the findings of CheckMate 142 also indicate that the integration of Nivolumab and Ipilimumab in first-line therapy has exhibited favorable clinical benefits (35). It is the performance of this regimen in first-line treatment for MSI that reinforces the value of dual ICIs in treating mCRC. On the other hand, Atezolizumab plus SOC exhibits the most potential therapeutic value in this subgroup. Although it demonstrates significant heterogeneity among MSI subgroups, given its remarkable therapeutic advantages in a limited number of MSI patients, our study considers that its high potential therapeutic value in MSI subgroups merits further exploration, and therefore also recommends its prioritized application in first-line therapy. Notably, despite pembrolizumab’s unremarkable superiority over SOC in terms of PFS and safety, it outperforms the control group in OS (Overall Survival, HR=0.72), ORR (Objective Response Rate, OR=1.66), and CR (Complete Response, OR=3.75) in the KEYNOTE-177 trial, hence qualifying as a robust recommendation as well.

And in the MSS subgroup, the results indicated that Nivolumab plus SOC represents the most promising approach, as it achieved the best therapeutic benefit in terms of PFS. Although the original studies lacked other efficacy assessment indicators and safety data for the MSS subgroup, the two original studies involved showed the following: in Checkmate9×8, where MSS patients accounted for 93% of all patients, the ORR (OR=1.78), CR (OR=3.33), and duration of response (DOR) ≥12 months (OR=2.4), OS (OR=1.03), and AE≥3 (OR=3.17) were reported; in the METIMMOX trial (all MSS patients), the OS (OR=0.75) and AEs≥3 (OR=0.78) were noted, with an OR value of only 0.49 for ORR, but six patients in the treatment group achieved CR, compared to none in the control group. It can thus be seen that Nivolumab plus SOC exhibits advantages in terms of direct tumor efficacy, albeit its safety requires further research and confirmation. Even though the prevailing belief is that MSS patients exhibit diminished sensitivity to ICIs as a consequence of primary drug resistance (36), which leads to unfavorable outcomes with ICIs, the findings of this study indicate that the use of ICIs as first-line therapy remains a beneficial approach for MSS patients.

In the second-line therapy, the results indicate that for the MSS subgroup, Atezolizumab plus SOC may be the optimal therapeutic option due to its significant advantage in PFS. The ORR data (OR=2.15) for the MSS subgroup was published in the BACCI study (86% MSS patients), showing the clinical value of this regimen despite the OS benefit (HR=0.96) in the overall population being just slightly higher than that of the control group. What is worth discussing is that although the benefit of Tremelimumab in combination with Durvalumab in PFS was not obvious in CO.26 (99% MSS patients), and its ORR rate was also low, it achieved considerable OS benefit (HR=0.72), which could directly extend the survival time of patients. Notwithstanding the high OS benefit, this treatment has a significantly higher incidence of adverse events than the comparator. Furthermore, it provides the least improvement in PFS among the other options. This discrepancy may be attributed to the mechanism of action of ICIs and the limited sample size in the original study. It should be noted that the alteration of the tumor microenvironment and the inhibition of immune evasion require time to take effect and given the progressive nature of the disease, patients receiving second-line treatment are often expected to have a shorter PFS. Moreover, research indicates that monotherapy is associated with more substantial gains in quality of life relative to combination therapy or no treatment (37). The elevated incidence of adverse effects observed with this regimen may be attributable to the combination of dual ICIs, which could be related to result in excessive T-cell activation (38) and the accumulation of toxicities (39). Overall, it is evident that due to the particularity of the refractory population, current second-line treatments for ICIs have varying strengths and weaknesses. Further evaluation is required to ascertain the best treatment options. Based on the existing research findings, clinicians are advised to consider patients’ treatment preferences when formulating treatment plans, and select the most suitable treatment options accordingly.

Despite the incomplete data observed in various studies, our subgroup data analysis yielded intriguing observations. Three studies (28–30) on first-line treatment (Nivolumab or Atezolizumab plus SOC) included patients of different ages in their analysis of PFS. For patients over the age of 60, the HRs in the AtezoTRIBE and METIMMOX trials were 0.59 (0.38–0.92) and 0.25 (0.07–0.93), respectively, while the HRs for patients over the age of 65 in Checkmate9×8 was 0.38 (0.17–0.86). These findings align with ours, where Nivolumab plus SOC not only demonstrated the most significant benefit for PFS in the MSS subgroup, but also appeared to provide superior clinical benefits to elderly patients, despite minor discrepancies in age groupings across the three studies. It has previously been demonstrated that tumor mutational burden(TMB) is an independent predictor of the efficacy of ICIs against various solid tumors (40, 41). Within the TMB subgroup, the HRs for those with high TMB was 0.80 (0.28-2.23) in CheckMate9×8 and 0.10 (0.02–0.51) in Atezolizumab. Of note, TMB levels in AtezoTRIBE patients were observed to be generally higher than those in Checkmate9×8, indicating that Atezolizumab in conjunction with SOC may offer more pronounced clinical benefits to patients exhibiting high TMB levels. It is plausible that the observed association between higher TMB and enhanced anti-tumor immunotherapy activity may in fact be causal (42). However, given the limited evidence currently available, further research and investigation are necessary to reach definitive conclusions.

Whether the combination of different chemotherapeutic drugs affects the efficacy of ICIs is also a topic worthy of discussion. In first-line treatment, three studies primarily targeting the MSS subgroup adopted FOLFOX6 as the basic chemotherapy regimen. Notably, CheckMate 9×8 incorporated Bevacizumab, while AtezoTRIBE further added irinotecan to this foundation. As an anti-VEGF agent, Bevacizumab combined with ICIs can not only inhibit tumor angiogenesis but also remodel the immunosuppressive tumor microenvironment, facilitating the recruitment and proliferation of immune cells, thereby promoting immune response (43). Irinotecan, a topoisomerase inhibitor, is classified among the essential chemotherapy options for colorectal cancer alongside oxaliplatin and others. Studies have shown that these drugs can stimulate tumor cells to release tumor antigens, upregulate co-inhibitory ligands such as PD-L1 in malignant cells or tumor-infiltrating lymphocytes, and enhance dendritic cell activation and PD-L1 expression, ultimately influencing the effects of ICIs. However, variations in the types, doses, administration methods, and sequential patterns of chemotherapeutic drugs may lead to distinct immunomodulatory effects (44). Therefore, extensive research data is required to confirm whether irinotecan usage impacts the efficacy of ICIs. In second-line therapy, BACCI adopts Bevacizumab plus capecitabine as the basic treatment regimen. Currently, capecitabine is a commonly used chemotherapeutic drug for colorectal cancer. Research has validated that single chemotherapeutic agents exhibit limited antitumor activity, while combination therapy effectively improves efficacy and reduces tumor resistance over the long term (45). Thus, further investigation is required to determine whether integrating ICIs with standard combination therapy regimens across different subgroups influences ICIs’ antitumor mechanisms.

Admittedly, there are some limitations to our study. Firstly, the number of RCTs included in this study is limited and the patient population is insufficient in number. Despite a comprehensive search of four major English databases, the scarcity of completed relevant studies limited the number of articles included to seven, five of which were phase II trials. It is therefore recommended that the findings of this study be validated by means of a larger sample size.

Secondarily, the study focused on endpoints in mCRC patients, lacking comprehensive exploration of individualized patient data. Furthermore, ICIs carry a substantial treatment cost for patients. Although elucidating their cost-effectiveness holds paramount significance for optimizing treatment protocols, clinicians must weigh this factor against practical scenarios given the disparities in drug costs across different regions, brands, and healthcare systems. Additionally, it is noteworthy that since the application of ICIs is still in its exploratory phase, the primary studies included in this research are relatively recent, and the PFS and safety indicators reported are based on short-term observations only. Thus, further research data are required to supplement and refine our understanding of their long-term efficacy and safety profiles.

Finally, due to limitations of the original studies, the current analysis of endpoints is insufficiently comprehensive. Most of the original studies did not comprehensively report the data of MSS and MSI subgroups. Consequently, the endpoints performance of the included studies with regard to the unreported subgroup in question remains uncertain.

In addition, subtle differences do exist among the seven studies with respect to their methodologies. For instance, in KEYNOTE-177, some patients switched from chemotherapy to Pembrolizumab during the course of therapy. In CO.26, the control group was administered best supportive care, a treatment measure that lacked specific descriptions. Consequently, it cannot be ascertained whether these factors will have an impact on the results of this study. In light of this uncertainty, further research and the collection of additional experimental data are essential.

## 5 Conclusion

A systematic review and meta-analysis of RCTs investigating ICIs as a treatment for mCRC revealed that ICIs offer advantages in two endpoints compared to SOC. This advantage is observed across both subgroups in first- and second-line treatments despite some manageable safety risks. This study indicates that ICIs have the potential to be a promising treatment option for MSS patients in the first-line setting. and Nivolumab plus SOC has emerged as a preferred option. In the MSI subgroup of first-line treatment, Nivolumab combined with ipilimumab has significantly improved PFS while ensuring the best safety, demonstrating its potential to become the optimal regimen for the MSI subgroup. The clinical advantages of Atezolizumab plus SOC require further verification, whereas pembrolizumab monotherapy is a relatively stable option.

However, the outlook for second-line therapy remains challenging. The current research allows clinicians to select relatively suitable regimens based on patients’ therapeutic needs. Specifically, in terms of the definite conclusion, Atezolizumab combined with SOC provides a higher PFS benefit, and also has an advantage in improving ORR in the original study. Tremelimumab plus Durvalumab is disappointing in improving PFS, but in the original study, it demonstrates a relatively significant improvement in OS, although the optimal regimen still requires further trial data and support from multiple samples.

## Data Availability

The original contributions presented in the study are included in the article/supplementary material. Further inquiries can be directed to the corresponding author.
